# Investigation of Bisphosphonate Therapy for Osteoporosis: A Single‐Center Cross‐Sectional Study

**DOI:** 10.1002/jgf2.70126

**Published:** 2026-05-13

**Authors:** Taku Harada, Shintaro Kosaka

**Affiliations:** ^1^ Department of General Medicine Nerima Hikarigaoka Hospital Tokyo Japan; ^2^ Department of Generalist Medicine Dokkyo Medical University Hospital Tochigi Japan; ^3^ Department of Hospital General Medicine Tokyo Metropolitan Hiroo Hospital Tokyo Japan

**Keywords:** atypical femoral fracture, bisphosphonates, care transition, osteoporosis

## Abstract

**Background:**

This study aimed to examine the management of bisphosphonate (BP) therapy for osteoporosis in Japan, focusing on treatment duration, dual‐energy X‐ray absorptiometry (DXA) assessment status, therapy despite renal impairment, and history of atypical femoral fracture (AFF).

**Methods:**

This single‐center, cross‐sectional study included 117 women inpatients aged ≥ 65 years admitted to the Department of General Medicine at Nerima Hikarigaoka Hospital between October 2022 and September 2025 while receiving BP therapy. The primary outcome was the prevalence of ultra‐long‐term therapy (≥ 10 years oral or ≥ 6 years intravenous). Secondary outcomes included long‐term therapy (≥ 5 years oral or ≥ 3 years intravenous), unknown treatment duration, inadequate DXA assessment, therapy despite renal impairment (serum creatinine ≥ 1.5 mg/dL), and AFF history.

**Results:**

The mean age was 84.3 ± 7.1 years. Ultra‐long‐term therapy occurred in 5.1% of patients, long‐term therapy in 23.1%, unknown treatment duration in 29.9%, and inadequate DXA assessment in 49.6%. Therapy despite renal impairment was noted in 6.8%, and one patient (0.8%) had AFF. Unknown treatment duration and inadequate DXA assessment were significantly more common in nonorthopedic care and in facility‐ or home‐based settings (*p* < 0.01).

**Conclusions:**

Among older women in Japan, ultra‐long‐term BP therapy was rare; however, nearly half lacked adequate assessment of treatment duration or documented DXA evaluation. These findings reveal shortcomings in long‐term osteoporosis management, particularly in nonorthopedic and facility‐ or home‐based care settings. Standardized reassessment, appropriate DXA evaluation, and improved information sharing across care transitions are essential to optimize treatment quality and safety in an aging population.

## 1 Introduction

Postmenopausal osteoporosis causes fractures more frequently than stroke, myocardial infarction, or breast cancer [1], representing a major public health issue [2]. Bisphosphonates (BP) reduce vertebral fracture incidence by 40%–70% and hip fractures by 20%–50%, and have long served as first‐line therapy for osteoporosis [3]. Although newer anabolic agents, including teriparatide, abaloparatide, and romosozumab, are recommended for patients at very high fracture risk, BPs remain the primary option because of their cost‐effectiveness, convenient administration, and extensive long‐term safety and efficacy data [2, 4]. However, atypical femoral fracture (AFF), a rare but serious adverse effect linked to prolonged BP use, has drawn growing concern. The risk of AFF increases with treatment duration [5], and rises markedly after ≥ 10 years of use.

Accordingly, the American Society for Bone and Mineral Research (ASBMR) recommends reassessing BP therapy after 5 years of oral or 3 years of intravenous treatment; continuation up to 10 years for oral bisphosphonates or 6 years for intravenous bisphosphonates may be considered in patients at high fracture risk [3].

A “drug holiday” refers to a planned temporary discontinuation of bisphosphonate therapy after an initial treatment period in patients at low to moderate fracture risk. This strategy is based on the long skeletal retention of bisphosphonates and aims to reduce the risk of rare adverse events, such as atypical femoral fracture, while maintaining residual anti‐fracture effects [3]. Recent reports indicate that AFF occurs particularly often in East Asian women [6], suggesting that long‐term BP–related AFF risk is also a significant concern in Japan.

In clinical settings, however, an evidence–practice gap persists. While underdiagnosis and undertreatment are well documented [1, 7, 8, 9], fewer studies have examined the reassessment of ongoing therapy, including treatment duration, DXA assessment, and decisions regarding discontinuation [10]. A recent study from Japan reported that only 13.9% of patients receiving osteoporosis treatment underwent DXA evaluation, and even among those treated with bisphosphonates, the rate was 41.4%, highlighting substantial gaps in bone mineral density assessment in clinical practice [11].

Because osteoporosis is highly prevalent among older adults and its management often extends across multiple care settings—from specialist to primary and home‐based care—standardized monitoring and reassessment are essential for ensuring treatment quality and safety. This study was designed to evaluate real‐world BP management among older women in Japan, focusing on treatment duration and inadequate DXA assessment. By revealing how frequently long‐term therapy continues without adequate reassessment and identifying settings where management is most suboptimal, this study aims to provide new insight into an underexplored aspect of osteoporosis care. These findings are expected to inform clinical and policy efforts to reduce the evidence–practice gap in long‐term osteoporosis management in Japan.

## 2 Methods

This single‐center, cross‐sectional observational study was conducted in the Department of General Medicine at Nerima Hikarigaoka Hospital, an acute care facility in Tokyo, Japan. The study period extended from October 1, 2022, to September 30, 2025. Consecutive women inpatients aged ≥ 65 years who were receiving BP therapy for osteoporosis, either orally or intravenously, were eligible.

### 2.1 Inclusion and Exclusion Criteria

Patients were included if hospitalized during the study period and confirmed to be receiving BP therapy for osteoporosis. Exclusion criteria were: (1) comorbidities requiring long‐term BP therapy that could affect bone metabolism (e.g., systemic steroid use, inflammatory bowel disease, malabsorption syndromes, chronic liver disease, or metastatic bone tumors) because these conditions may differ from primary osteoporosis in pathophysiology and management; (2) insufficient treatment information, defined as the inability to obtain necessary data on treatment duration or DXA assessment due to nonresponse to institutional inquiries; and (3) multiple admissions of the same patient, for which only the first hospitalization was analyzed.

### 2.2 Data Collection

Data were extracted from medical records and patient medication notebooks by the attending physician or ward pharmacist. For patients identified as receiving BP therapy, structured written inquiries were sent to prescribing institutions (by fax or mail) to confirm treatment duration and determine whether any dual‐energy X‐ray absorptiometry (DXA) examination had been performed before or during bisphosphonate therapy. Cases with nonresponse to institutional inquiries, resulting in unavailable information on treatment duration or DXA assessment, were excluded from the analysis.

### 2.3 Variables

Collected variables included patient demographics (age), comorbidities, renal function, cognitive impairment, activities of daily living (ADL), frailty, long‐term care certification, BP type and initiation date, prescribing department, and prescribing institution. Comorbidities were quantified using the Charlson Comorbidity Index (CCI) [12]. Frailty was assessed with the Clinical Frailty Scale (CFS), categorized into nine levels, and classified according to previously validated criteria [13, 14, 15, 16]. Cognitive impairment was defined as the presence of a documented diagnosis of dementia in the medical record or a Mini‐Mental State Examination (MMSE) score of < 24 when available. MMSE was not systematically performed in all patients. ADL was recorded as independent ambulation, assisted ambulation, or inability to walk. Long‐term care certification was defined as certification under the Japanese public long‐term care insurance system (Kaigo Hoken), indicating eligibility for support or care services based on functional and cognitive impairment. Unlike mobility‐based ADL indicators such as independent ambulation, this certification reflects a comprehensive assessment that includes physical, cognitive, and social care needs.

### 2.4 Drug Classification and Renal Function

Evaluated BPs included oral agents approved for osteoporosis in Japan (alendronate, risedronate, minodronate, ibandronate) and intravenous agents (ibandronate, zoledronate). Renal impairment was defined as sustained serum creatinine (Cr) ≥ 1.5 mg/dL, approximately corresponding to an eGFR of 28 mL/min/1.73 m^2^ in a 65‐year‐old woman. Cases in which BP therapy continued despite renal impairment were identified. Pre‐hospitalization renal data were supplemented from previous hospital records and primary care physician reports. This definition was used solely for assessment and classification, not for inclusion.

### 2.5 Clinical Setting Classification

Prescribing departments were grouped as (i) internal medicine (including general medicine, internal medicine subspecialties such as endocrinology/metabolism and rheumatology), (ii) orthopedics, and (iii) other specialties, defined as departments not primarily involved in osteoporosis management or bone‐related care (e.g., surgery, psychiatry, anesthesiology, radiology, pathology). Prescribing institutions were categorized as clinics or hospitals, and care settings as outpatient, home/facility‐based, or “other” (including initiation during hospitalization or rehabilitation).

### 2.6 Study Outcomes

The primary outcome was the proportion of patients receiving ultra‐long‐term BP therapy, defined as ≥ 10 years of oral or ≥ 6 years of intravenous therapy [3]. Secondary outcomes were: (1) the proportion receiving long‐term therapy (≥ 5 years oral or ≥ 3 years intravenous); (2) the proportion with unknown treatment duration; (3) the proportion with inadequate DXA assessment, defined as the absence of any documented dual‐energy X‐ray absorptiometry (DXA) examination before or during bisphosphonate therapy; (4) the proportion continuing BP therapy despite renal impairment; and (5) the proportion with a history of atypical femoral fracture (AFF), verified by attending physicians through medical records and referral letters. Patients meeting any of the following conditions—ultra‐long‐term therapy, unknown duration, inadequate DXA assessment, or therapy despite renal impairment—were also classified as having “potential suboptimal management.”

### 2.7 Statistical Analysis

Categorical variables were compared using the chi‐square or Fisher's exact test, as appropriate, and continuous variables were analyzed with the Student's *t*‐test or Wilcoxon rank‐sum test, depending on distribution. Normality was evaluated with the Shapiro–Wilk test. All analyses were conducted using EZR (Easy R) [17]. Two‐sided *p* values < 0.05 were considered statistically significant.

### 2.8 Use of Artificial Intelligence

ChatGPT (OpenAI) was used solely to assist with language editing of the manuscript, including improvement of grammar, sentence clarity, readability, and overall English expression. It was not used for study design, data collection, data analysis, statistical analysis, interpretation of the results, or formulation of the scientific conclusions. All text was reviewed and revised by the authors, who take full responsibility for the accuracy and integrity of the manuscript.

## 3 Ethics

The study protocol was approved by the Ethics Committee of Nerima Hikarigaoka Hospital (approval number: 22082501). An opt‐out consent process was implemented through public notices within the hospital and on the institutional website. The study complied with the Declaration of Helsinki. As this was an exploratory observational study of real‐world practice, no formal sample size calculation was performed.

## 4 Results

**FIGURE 1:**
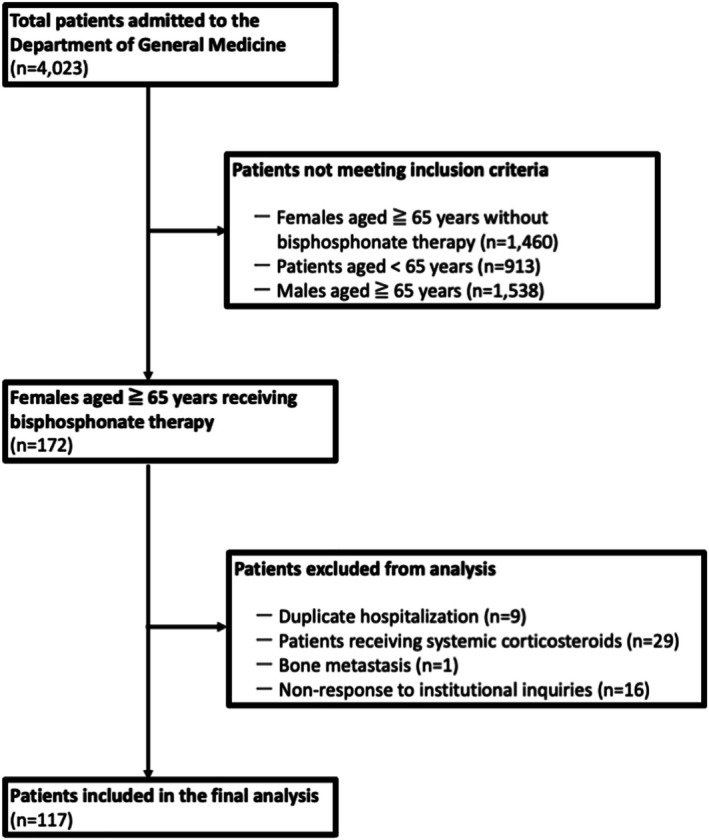
Patient flow for inclusion in the study. Among 4023 patients admitted to the Department of General Medicine, 172 women aged ≥ 65 years receiving bisphosphonate therapy were screened. Fifty‐five patients were excluded due to duplicate hospitalizations, systemic corticosteroid use, bone metastasis, or nonresponse to institutional inquiries. The final analysis included 117 patients.

During the study period, 4023 patients were admitted to the Department of General Medicine. Among them, 172 women aged ≥ 65 years were receiving BP therapy. After excluding duplicate hospitalizations (*n* = 9), patients with steroid use (*n* = 29), one case prescribed BP for metastatic bone tumor (*n* = 1), and those with nonresponse to institutional inquiries (*n* = 16), 117 patients were included in the final analysis (Figure 1). The mean age was 84.3 ± 7.1 years, the mean Charlson Comorbidity Index was 1.5 ± 1.2, and the mean Clinical Frailty Scale score was 5.1 ± 1.0. Cognitive impairment was identified in 49 patients (41.9%), and nearly half (49.6%) required long‐term care certification. Regarding mobility, 29 patients (24.8%) required assisted ambulation, and 30 (25.6%) were unable to walk independently (Table 1). These findings indicate that the study cohort primarily comprised frail older women with multiple comorbidities and limited mobility. Oral BPs predominated: alendronate in 50 patients (42.7%), minodronate in 33 (28.2%), risedronate in 19 (16.2%), and ibandronate in five (4.3%). Intravenous agents were less common—ibandronate in nine patients (7.7%) and zoledronate in one (0.9%). Prescriptions originated mainly from internal medicine departments (47.9%), followed by orthopedics (37.6%) and other specialties (14.5%). Regarding institutional type, 78.6% of prescriptions were issued by clinics and 21.4% by hospitals. Most patients were managed in outpatient clinics (71.8%), while 24.8% received facility‐ or home‐based care and 3.4% initiated therapy during hospitalization or rehabilitation.

**TABLE 1:** Baseline clinical characteristics of older women receiving bisphosphonate therapy for osteoporosis.

Characteristics	Total (*n* = 117)
Age	84.3 ± 7.1
Charlson Comorbidity Index	1.5 ± 1.2
Clinical Frailty Scale	5.1 ± 1.0
Cognitive impairment	49 (41.9%)
Independent ambulation	58 (49.6%)
Assisted ambulation	29 (24.8%)
Unable to walk independently	30 (25.6%)
Physician characteristics	
Orthopedics	44 (37.6%)
Internal medicine	56 (47.9%)
Other specialties	17 (14.5%)
Healthcare facility characteristics	
Hospital	25 (21.4%)
Clinic	92 (78.6%)
Care setting	
Outpatient care	84 (71.8%)
Facility‐ or home‐based care	29 (24.8%)
Other care setting	4 (3.4%)
Primary outcome	
Ultra‐long‐term bisphosphonate therapy	6 (5.1%)
Secondary outcomes	
Long‐term bisphosphonate use	27 (23.1%)
Unknown treatment duration	35 (29.9%)
Inadequate DXA assessment	58 (49.6%)
Renal impairment	8 (6.8%)
Atypical femoral fracture	1 (0.8%)
Potential suboptimal management	76 (65.0%)

For the primary outcome, 6 patients (5.1%) were receiving ultra‐long‐term BP therapy (≥ 10 years oral or ≥ 6 years intravenous). Among secondary outcomes, 27 patients (23.1%) had long‐term therapy, 35 (29.9%) had unknown treatment duration, and 58 (49.6%) had inadequate DXA assessment, indicating that no documented DXA examination had been performed before or during bisphosphonate therapy in about half the cohort. Eight patients (6.8%) continued therapy despite renal impairment (serum Cr ≥ 1.5 mg/dL), including two with end‐stage renal failure. Only one patient (0.8%) had a history of atypical femoral fracture (Table 2). Subgroup analysis showed that unknown treatment duration and inadequate DXA assessment were significantly more common in internal medicine and other specialties than in orthopedics (both *p* < 0.001). Consequently, the prevalence of potential suboptimal management was also significantly higher in these departments (*p* = 0.008) (Table 2). Comparisons between hospitals and clinics revealed no significant differences (Table 3). However, notable disparities appeared by care setting: patients in facility‐ or home‐based care had significantly fewer long‐term BP users (3.4% vs. 31.0%, *p* = 0.002) but markedly higher rates of unknown treatment duration (65.5% vs. 19.0%, *p* < 0.001), inadequate DXA assessment (82.8% vs. 38.1%, *p* < 0.001), and potential suboptimal management (89.7% vs. 57.1%, *p* = 0.001) compared with those managed in outpatient clinics (Table 4).

**TABLE 2:** Management outcomes of bisphosphonate therapy stratified by prescribing department (orthopedics, internal medicine, and other specialties).

	Orthopedics (*n* = 44)	Internal medicine (*n* = 56)	Other specialties (*n* = 17)	*p*
Ultra‐long‐term bisphosphonate use	3	2	1	0.629
Long‐term bisphosphonate use	14	11	1	0.0485
Unknown treatment duration	4	23	8	< 0.001
Inadequate DXA assessment	3	4	1	1
Renal impairment	10	35	13	< 0.001
Atypical femoral fracture	1	0	0	0.521
Potential suboptimal management	21	41	14	0.008

**TABLE 3:** Management outcomes of bisphosphonate therapy stratified by institutional type (clinics vs. hospitals).

	Clinic (*n* = 92)	Hospital (*n* = 25)	*p*
Ultra‐long‐term bisphosphonate use	5	2	0.641
Long‐term bisphosphonate use	25	2	0.059
Unknown treatment duration	28	7	1
Inadequate DXA assessment	6	2	0.679
Renal impairment	49	9	0.176
Atypical femoral fracture	0	1	0.214
Potential suboptimal management	62	14	0.346

**TABLE 4:** Management outcomes of bisphosphonate therapy stratified by care setting (outpatient vs. facility‐ or home‐based care).

	Outpatient care (*n* = 84)	Facility‐ or home‐based care (*n* = 29)	*p*
Ultra‐long‐term bisphosphonate use	6	0	0.336
Long‐term bisphosphonate use	26	1	0.002
Unknown treatment duration	16	19	< 0.001
Inadequate DXA assessment	5	3	0.421
Renal impairment	32	24	< 0.001
Atypical femoral fracture	1	0	1
Potential suboptimal management	48	26	0.001

## 5 Discussion

This single‐center cross‐sectional study of women aged ≥ 65 years admitted to an acute care hospital provides important insights into real‐world BP therapy management for osteoporosis in Japan. Although ultra‐long‐term therapy (≥ 10 years) was uncommon (5.1%), a considerable proportion of patients had unknown treatment duration (29.9%) or inadequate DXA assessment (49.6%). These findings reveal major deficiencies in ongoing BP therapy evaluation, especially in nonorthopedic departments and facility‐ or home‐based care, indicating that long‐term osteoporosis management may be insufficiently maintained.

This study complements earlier work by identifying an evidence–practice gap in this underexamined area. Cases with unknown treatment duration or inadequate DXA assessment were significantly more frequent in internal medicine and other specialties than in orthopedics (both *p* < 0.001). Compared with Canadian registry data, where > 60% of patients continued BP therapy for > 5 years and 40% for > 10 years [10], the long‐term treatment rate in this study was markedly lower. This difference likely reflects methodological contrasts: registry and pharmacy data allow precise prescription tracking, whereas in clinical practice, physicians often lack detailed knowledge of prior treatment histories. Consequently, many patients were categorized as “unknown.” These findings may accurately represent Japan's clinical reality, where indefinite therapy continuation often occurs without systematic reassessment or documentation.

Among six patients who received BP therapy for > 10 years, two had no fracture history but YAM (young adult mean) < 70%. Three were treated for fragility fractures, such as vertebral or proximal femoral fractures, where therapy continuation may have resulted from shared decision making. One patient without fracture history and YAM ≥ 70% may have been a candidate for discontinuation of therapy [3, 4]. The 2015 Japanese guidelines allowed continuation for 3–5 years but lacked explicit recommendations for longer use [18], unlike international guidance, which recommends reassessment after 5 years of oral or 3 years of intravenous therapy, with continuation up to 10 and 6 years, respectively, considered in selected high‐risk patients [3]. This ambiguity likely contributed to variability in clinical practice. Given that atypical femoral fracture (AFF) risk is notably high in East Asian women [6], regular evaluation of therapy duration is particularly important in Japan.

The high prevalence of inadequate DXA assessment in nearly half the patients highlights gaps in treatment evaluation. This finding is consistent with a recent Japanese study, which reported that only 41.4% of patients treated with bisphosphonates underwent DXA evaluation, supporting the presence of substantial gaps in bone mineral density assessment in clinical practice [11]. Although earlier guidelines, such as those from the American College of Physicians [19], recommended against serial DXA testing, recent updates emphasize regular reassessment [20], consistent with other international recommendations [2, 3, 21]. The 2015 Japanese guidelines did not specify testing intervals but endorsed DXA evaluation, a recommendation reaffirmed in the 2025 revision [4, 18]. In the 2025 revision, bone mineral density assessment using DXA is recommended approximately 1 year after treatment initiation, and thereafter at intervals of 1 year or longer once the treatment regimen has been established [4]. Nonetheless, clinical implementation remains limited, possibly due to restricted access to equipment, low awareness among nonorthopedic physicians, and limited reimbursement incentives. Because adherence to BP therapy is often suboptimal [3], appropriate DXA assessment is essential to verify treatment efficacy and guide continuation or discontinuation.

Physician education and care coordination may also influence management quality. In Japan, few physicians receive formal primary care training, and many clinic‐based practitioners transition from hospital specialties without structured preparation [22]. Consequently, awareness of guideline updates and long‐term management strategies may be insufficient. Moreover, information transfer during transitions to long‐term care or home care is often inadequate, contributing to high rates of unknown treatment duration and inadequate DXA assessment. Previous studies have shown that structured communication during care transitions improves treatment continuity and reduces undertreatment [23], suggesting the need for similar systems in Japan.

Overall, beyond underdiagnosis and undertreatment [7, 8], this study identifies inadequate reassessment of BP therapy as a further contributor to suboptimal osteoporosis management. Improvement will require systemic strategies such as standardized recording of treatment duration in medical records or medication notebooks, integration of routine DXA assessment during therapy into care pathways, and effective communication systems across care transitions. In Japan's aging society, strengthening osteoporosis management is vital to fracture prevention and the reduction of frailty and long‐term care dependence.

The strengths of this study include data from more than 80 medical institutions and detailed assessment of treatment duration, DXA assessment, and care setting. However, this study has several limitations. First, as a single‐center study in an urban acute care hospital, generalizability may be limited, and the inpatient population likely had greater frailty than the broader outpatient population. Second, data collection partly depended on reports from prescribing institutions, and nonresponse to institutional inquiries resulted in exclusion of some patients due to unavailable treatment information. Third, patients with conditions affecting bone metabolism or requiring bisphosphonate therapy for reasons other than primary osteoporosis, such as systemic steroid use or metastatic bone disease, were excluded to maintain a more clinically homogeneous study population; therefore, the findings may not be fully generalizable to patients with secondary osteoporosis or other nonprimary indications for bisphosphonate therapy. Fourth, because this study included only patients who were actively receiving bisphosphonate therapy at the time of hospitalization, patients who had temporarily discontinued treatment as part of a drug holiday were not captured. Therefore, the appropriateness of long‐term bisphosphonate use in relation to drug holidays could not be evaluated. Fifth, the reasons for therapy continuation or discontinuation and shared decision‐making processes could not be assessed. Future multicenter studies, including qualitative analyses, are required to clarify these determinants.

## 6 Conclusion

This study elucidates the current status of BP therapy among older women in Japan, showing that although ultra‐long‐term use was uncommon, many had unknown treatment durations and had inadequate DXA assessment, particularly in nonorthopedic and facility‐based care. These findings emphasize the need to strengthen reassessment practices, ensure routine bone mineral density assessment, and improve information sharing during care transitions to enhance the quality of long‐term osteoporosis management in Japan.

## Author Contributions

**Shintaro Kosaka:** writing – review and editing, project administration, supervision, investigation, validation. **Taku Harada:** conceptualization, methodology, data curation, investigation, validation, formal analysis, visualization, project administration, resources, writing – original draft.

## Funding

The authors have nothing to report.

## Ethics Statement

The study protocol was approved by the Ethics Committee of Nerima Hikarigaoka Hospital (approval number: 22082501). The study complied with the Declaration of Helsinki. As this was an exploratory observational study of real‐world practice, no formal sample size calculation was performed.

## Consent

An opt‐out consent process was implemented through public notices within the hospital and on the institutional website.

## Conflicts of Interest

The authors declare no conflicts of interest.

## Data Availability

The datasets generated and/or analyzed during the current study are available from the corresponding author on reasonable request.
